# Incidence of seriously injured road users in a Swedish region, 2003–2014, from the perspective of a national road safety policy

**DOI:** 10.1186/s12889-019-7937-0

**Published:** 2019-11-27

**Authors:** Astrid Värnild, Peter Larm, Per Tillgren

**Affiliations:** 1 School of Health, Care and Social Welfare, Mälardalen University, Box 883, SE-721 23 Västerås, Sweden; 2 Department of Clinical Neuroscience, Karolinska Institutet, SE-171 77 Stockholm, Sweden

**Keywords:** Vision Zero, Policy, Road injury, STRADA, ISS, Rural, Urban, Incidence

## Abstract

**Background:**

Since 1997 Sweden has a policy for road safety called Vision Zero. Given that Vision Zero is mainly used to reduce fatalities among car occupants, the question has been raised by the research community whether a Vision Zero approach promotes health for all road traffic users. The objective is to measure target fulfilment of the national road safety policy for a Swedish region by examining incidence of serious injury during 2003–2014 in rural and urban road spaces with or without implemented measures.

**Methods:**

Data on seriously injured road users, defined as ISS > 8 (Injury Severity Score), were retrieved from STRADA (Swedish Traffic Accident Data Acquisition) together with data from NVDB (National Road Database). These data are used to describe where road users are seriously injured in relation to implemented national policy and using a conceptual model of a road space comprising roads, pavements and tracks for walking and cycling. Seriously injured road users in single and multiple crashes with and without vehicles are included. The development of the incidence is analysed for different road users and places in the road space.

**Results:**

Despite implemented road safety measures in the region, the incidence of seriously injured road users per 100,000 inhabitants in rural areas increased from 7.8 in 2003 to 9.3 in 2014 but doubled in urban areas from 8.0 in to 16.3 respectively. In areas not transformed by Vision Zero, only 36% were injured in rural areas while 64% were injured in urban areas. In contrast, in transformed areas 61% of injuries occurred in rural areas, whereas 39% occurred in urban areas. While the incidence decreased for car occupants on transformed national roads in rural areas, the incidence of serious injuries increased among unprotected road users in urban areas, in particular on pavements and tracks for cycling and walking than on the roads where Vision Zero had been implemented.

**Conclusion:**

The reduction in the incidence for car occupants in the region may not be adequate to contribute to fulfilling the national target. More needs to be done, especially in the urban areas, where more active mobility is desired.

## Background

Road traffic injuries are a major public health issue. In 2013, all injuries accounted for 10.1% of the global burden of disease. Approximately a third of years of life lost by injuries were due to road injuries [1]. Each year, about 1.35 million people are killed in road traffic and about 50 million more are injured, many of whom remain disabled for life [2]. The United Nations Sustainable Development Goals from 2015 include two targets for safer road traffic: halving the number of global deaths and injuries by 2020 and improving road safety in cities by 2030 [3]. More than half of all serious injuries in the EU occur in urban areas, and especially affect pedestrians and other vulnerable road users [4]. European statistics indicate that single pedestrian and bicycle crashes are a more important issue than previously anticipated, and it can be expected that the share of unprotected road users will increase because of ageing populations and urbanization [5]. An increased use of individual cars in industrialized countries has allowed urban areas to sprawl and made walking and cycling less feasible, disadvantaging people who live without cars such as women, children, older people and people with disabilities [3, 6].

Multiple road safety management approaches have been developed, however there is no standard package for road safety interventions suitable for all contexts and countries [7]. The Swedish model of preventing injuries, referred to as Vision Zero, has the long-term goal of zero fatalities and serious injuries in road traffic, and aims to adapt the design and function of the road transport system to meet this goal [8–11].

In road injury epidemiology, ideas, findings and control measures are based on distinguishing between three factors: human, vehicle and environment [12]. These factors are incorporated as potentially safe components in a model developed by the Swedish Road Administration, and have been introduced in Swedish road safety measures. The model is related to the requirements of international programmes for car and road safety assessments, and is premised on the idea of a safe road user who wears a seat belt, does not exceed the speed limit and is sober. It specifies the biomechanical limits for what the road user can tolerate without sustaining serious injury. Speed limits play a fundamental role in the model. The safety level must increase if the speed limit increases [13].

In Sweden the efforts to reconstruct roads in accordance with Vision Zero began with the introduction of median barriers in 1997; this was followed by speed-camera sections of road in 2006, and in 2009 by a national revision of speed limits on rural roads combined with a new speed system for both rural (national and regional) and urban roads. Barrier-separated roads and speed camera sections have been implemented on national roads, but not on regional roads (Table 1). On urban roads, implementation of the new speed limit system started after 2009 and is still ongoing, but local authorities have been allowed to reduce the speed limit to 30 km/hour on individual streets since 1998. Since that time efforts have also been underway to reduce the effects of kinetic energy [12] in specific locations by building roundabouts and adapting crossings for pedestrians and cyclists to a speed limit of 30 km/hour, but no safety measures have been initiated in road areas without speed limits such as pavements and tracks for walking and cycling.

**Table 1:** Facts about rural roads in Sweden and Region Västmanland [13, 14]

Rural roads	Sweden				Region Västmanland			
	Length km 2014	Speed revision 2009		Million vehicle km 2014	Length km 2014	Speed revision 2009		Million vehicle km 2009
		Increased speed 10 km/h	Decreased speed 10 km/h			Increased speed 10 km/h	Decreased speed 10 km/h	
National roads	15,600	1000	2500	37,000	362	61	98	1045
*Motorway*^a^	*2220*			*18,000*	*73*			
*Barrier separated road*^a^	*2790*			*4000*	*192*			
*Speed camera section*^a^	*3300*				*45*			
Regional roads	82,900	1650	15,350	21,000	1772	0	290	461
Total rural roads	98,500	2650	17,850	58,000	2134	61	388	1506

^a^Included in national roads

Given that parts of Vision Zero are implemented by regional authorities, however, the regional perspective is of interest, and fulfilment of the national road safety policy needs to be addressed even on a regional and local level. The state, together with the regions, is responsible for rural roads, while municipalities are responsible for urban roads. These parties are jointly responsible for achieving the road safety target. Vision Zero is mainly used to reduce fatalities among car occupants [13, 15–17]. Thus, the question has been raised by the research community whether a Vision Zero approach promotes health for all road traffic users [18]. From a public health perspective (Figs. 2 and 3), injuries of unprotected road users are of interest since their share in traffic will increase because of ageing populations and urbanization, but also because of the interest in various kinds of more active mobility in society [5, 6, 19, 20]. National data on crashes and injuries in road traffic from emergency hospitals in Sweden were not available until 2016/2017, and only two regions, including Region Västmanland, have data from 2000. Thus, this data provides a unique opportunity to study the development of injury rates when the national road safety policy was implemented on a regional and local level. The objective of this study is to measure the target fulfilment of a national road safety policy for a region by examining the incidence (number per 100,000 inhabitants) of seriously injured road users annually during 2003–2014 in rural and urban areas with or without implemented road safety measures.

The region setting consists of Västmanland (RV), one of Sweden’s 21 regions located near Stockholm, the capital city, with a population of 260,000 inhabitants in ten municipalities [21]. This represents 2.68% of Sweden’s total population of 9.7 million in 2014. During the study period, the proportion of the population living in urban area increased from 86 to 88% [22, 23]. RV is one of eighteen regions outside the three metropolitan regions; such regions consist of a central municipality together with a number of smaller municipalities. These regions contain 48% of Sweden’s population [21]. Comparisons of road characteristics and safety management activities between RV and the national level are provided in Table 1. The RV is crossed by six national roads with 69% of travelled kilometres on rural roads (Table 1). During the period 2003–2014, the number of travelled kilometres on motorways and barrier-separated roads increased from around 20 to 50% of the transports on national roads. Permanent speed cameras were introduced from 2006 and, together with speed-limit revision, 88.4% of the length of national roads in RV was transformed by Vision Zero in 2014. In 2009 the speed limit on regional roads with 31% of travelled kilometres was reduced from 90 to 80 km/h along 16% of their length (Table 1). Since 1998, municipalities in RV have decreased the speed-limit in urban areas to 30 km/h, but this process is still ongoing together with the introduction of 40 km/h from 2010.

## Method

### Data from registries, definition of serious injury, and study design

Data about road traffic injuries of all patients in emergency hospitals are reported to the STRADA registry (Swedish Traffic Accident Data Acquisition) [24], not only of patients who are hospitalized. Data about injuries in the registry are related to the location where the crash occurred. For this study we have defined a road space consisting of roads, pavements and tracks for walking and cycling and data about where in the road space the crash occurred has been retrieved from STRADA. Then data from STRADA has been examined in relation to data from NVDB (National Road Database) [14], and it is determined whether or not the location was transformed by Vision Zero measures at the time of the crash.

Health care data reported to the STRADA registry are linked to the AIS-scale (Abbreviated Injury Scale), which is constructed to determine and quantify outcomes of injuries [25]. The effect of one or more injuries on a person is calculated as an Injury Severity Score (ISS). The ISS value is the sum of the squares of the highest AIS-value of three out of six different body parts of a person. The definition of serious injury in this study is the same as STRADA’s definition of a seriously injured road user, ISS > 8.

### Variables

In the study, variables are linked to road user group, where the crash occurred, and when it happened. The road user is also defined by age and sex and whether it was a single or multiple crash; a crash with anyone else in the road space. The road user groups include pedestrians and persons using a vehicle such as a bicycle, moped, motorbike, car, lorry or bus. Types of areas are roads with different characteristics, tracks for pedestrians and cyclists [26] and pavements. Only persons injured in one of these areas have been included. Included in roads are also such areas as city squares and other facilities with mixed traffic. Six areas transformed under Vision Zero were defined together with five areas not transformed (Table 2).

**Table 2:** Serious injuries (ISS > 8) in Region Västmanland in rural and urban areas 2003–2014 (*N* = 633)

Variables	Rural area		Urban area		*p*-value
	*n* = 262	%	*n* = 371	%	^*Chi2*^-value
					df
Road user group					< 0.001
*Car or bus driver/passenger, lorry driver*	183	69.8%	32	8.6%	317.167
*Cyclist*	24	9.2%	161	43.4%	4
*Moped rider*	9	3.4%	22	5.9%	
*Motorcycle rider*	29	11.1%	9	2.4%	
*Pedestrian*	17	6.5%	147	39.6%	
Road area					< 0.001
*Vision Zero Area:*	83	31.7%	53	14.3%	27.540
Motorway 110 km/h	19		0		
Barrier separated road 100 km/h	27		0		
Speed camera section	17		0		
Road 80, 60 or 40 km/h	14		3		
Roundabout	6		2		
Road 30 km/h, over or under pass	0		48		
*Non-Vision Zero Area:*	179	68.3%	318	85.7%	
Road 110, 100, 90, 70 or 50 km/h	135		60		
Junction - T- or X-junction	35		37		
Pedestrian crossing, road ≥40 km/h	5		24		
Track for cyclist, pedestrian, moped rider	2		102		
Pavement	2		95		
Type of accident					< 0.001
*Multi crash*	131	50.0%	114	30.7%	24.041
*Single crach*	131	50.0%	257	69.3%	1
Age					< 0.001
*0–14*	12	4.6%	16	4.3%	68.641
*15–24*	50	19.1%	42	11.3%	5
*25–44*	76	29.0%	38	10.2%	
*45–64*	67	25.6%	98	26.4%	
*65–74*	32	12.2%	68	18.3%	
*75 and above*	25	9.5%	109	29.5%	
Sex					0.012
*Men*	171	65.3%	205	55.3%	6.381
*Women*	91	34.7%	166	44.7%	1

According to Vision Zero the designers of the system are responsible for the design, maintenance and use of the road transport system, and hence for the level of safety [11]. Therefore the study includes not only the road, but also pavements and tracks for cycling and walking, as well as their side areas [11, 27]. These elements form the road space as defined for this analysis. Within the road space, the crash occurs in a specified area that has or has not been transformed by road safety measures at the time of the crash. A transformed area ought to be safer than an area not transformed, but even in transformed areas people are killed or seriously injured. Road users in the road space may or may not have been using a vehicle in the crash that caused the injury. The designer/road authority (state in rural roads and municipalities in urban roads) has had the opportunity to implement road safety measures in the road space during the period.

### Analysis

The incidence rate, measured as number of seriously injured road users per 100,000 inhabitants for each year of the period, has been calculated. The trends are shown by linear regression for rural and urban areas, but also for car occupants and unprotected road users in different parts of the road space. Further, significant differences in serious injuries between rural and urban areas, and between Vision Zero and Non-Vision Zero Areas have been calculated with chi-square tests.

## Results

**Fig. 1:**
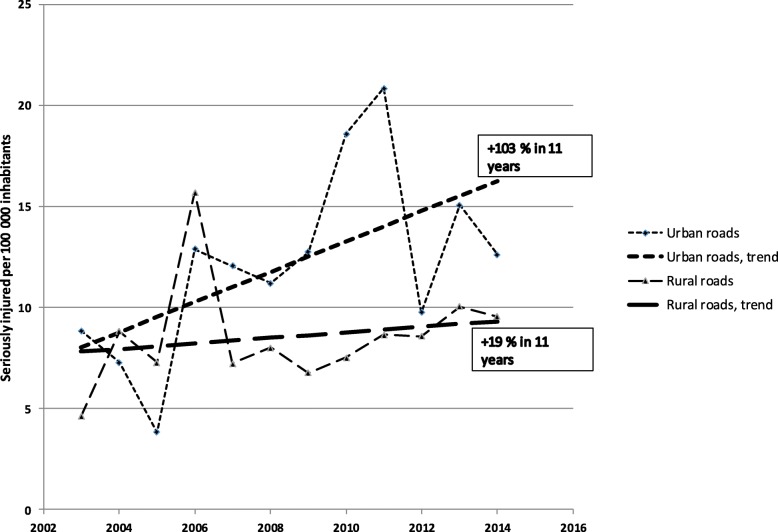
Serious injuries per 100,000 inhabitants on rural and urban roads in Region Västmanland 2003–2014

**Fig. 2:**
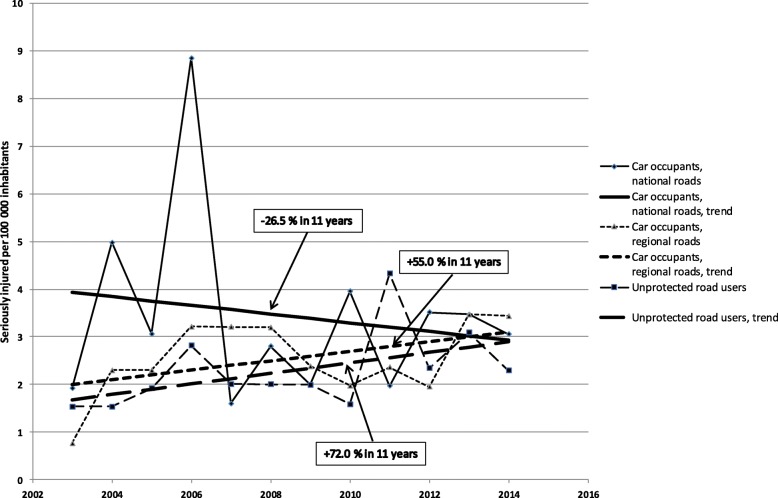
Serious injuries per 100,000 inhabitants on national and/or regional rural roads in Region Västmanland 2003–2014

**Fig. 3:**
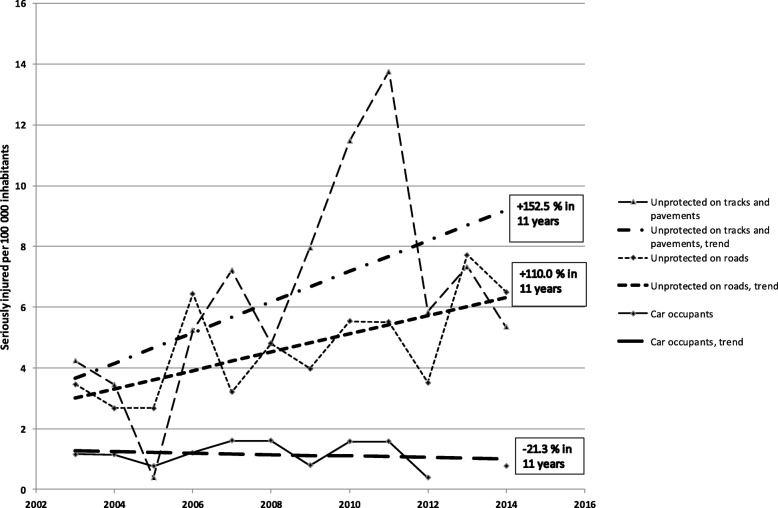
Serious injuries per 100,000 inhabitants on urban tracks and pavements or roads in Region Västmanland 2003–2014

The incidence increased for all seriously injured persons in rural areas from 7.8 per 100,000 inhabitants in 2003 to 9.3 in 2014, and for urban areas from 8.0 to 16.3 (Fig. 1). Despite implemented measures, there is a slight increase of incidence in rural areas, as compared to the doubling in urban areas. In particular, as shown in Fig. 2, the incidence for car occupants in rural areas increased by 55.0% on regional roads but dropped by 26.5% on national roads. Similarly, the incidence for car occupants in urban areas decreased by 21.3% (Fig. 3). In contrast, the incidence for unprotected road users in rural areas increased by 72.0% (Fig. 2). Further, the incidence for unprotected road users in urban areas increased by 110.0% on roads and by 152.5% on pavements and on cycle and pedestrian tracks (Fig. 3).

For all variables there are significant differences in the distribution of serious injuries between rural and urban areas (Table 2). In rural areas, 262 road users were injured, most of whom were car or bus drivers/passengers, lorry drivers or motorcycle drivers, 80.9%. Further, half of them were injured on roads using the old speed system and in multi-crash accidents. The majority, 65.3%, were men, and almost a third were in the age range 25–44. Furthermore, 83 were seriously injured in Vision Zero areas and 179 in non-Vision Zero areas. In urban areas, 371 road users were injured. Most of them, 83%, were cyclists or pedestrians, and over half, 53.1%, were injured on tracks for cyclists and pedestrians and on pavements. The majority, 55.3%, were males, and approximately one-quarter were in the age range 45–64. Further, 53 were seriously injured in Vision Zero areas and 318 in non-Vision Zero areas.

Comparisons between serious injuries in Vision Zero Areas and Non-Vision Zero Areas (Table 3) revealed that 21.5% of the injuries occurred in Vision Zero Areas and 78.5% in Non-Vision Zero Areas. Further, injured persons were on average 5.45 years older in areas not transformed by Vision Zero than in transformed areas. Half of the seriously injured persons in Vision Zero Areas were car drivers and 38.2% were pedestrians or cyclists, whereas in Non-Vision Zero Areas 29.6% were car drivers and 59.8% were pedestrians or cyclists. In non-transformed areas only 36% were injured in rural areas while 64% were injured in urban areas. In contrast, in transformed areas 61% of injuries occurred in rural areas whereas 39% occurred in urban areas.

**Table 3:** Distribution of serious injuries in Vision Zero Areas (*n* = 136) and Non-vision Zero Areas (*n* = 497) respectively

	Vision Zero Areas	Non-Vision Zero Areas	Difference statistics	*df*	*p-value*
Demographic characteristics					
*Men*	88 (64.7%)	288 (57.9%)			
*Women*	48 (35.3%)	209 (42.1%)	2.022	1	.155
*Age (mean age and SD)*	47.62 (22.03)	53.07 (23.64)	5.837	1	.016
Road user group					
*Pedestrian*	23 (16.9%)	141 (28.4%)	7.304	1	.007
*Cyclist*	29 (21.3%)	156 (31.4%)	5.229	1	.022
*Moped rider*	4 (2.9%)	27 (5.4%)			.271
*Motorcycle rider*	12 (8.8%)	26 (5.2%)	2.442	1	.118
*Car, bus or lorry driver/passenger*	68 (50.0%)	147 (29.6%)	19.857	1	< .001
Single or multiple crash					
*Single crash*	74 (54.4%)	314 (63.2%)			
*Multiple crash*	62 (45.6%)	183 (36.8%)	3.460	1	.063
Road area					
*Rural area*	83 (61.0%)	179 (36.0%)			
*Urban area*	53 (39.0%)	318 (64.0%)	27.540	1	< .001

Note. Difference statistics are estimated using chi-square tests reporting chi-square statistics with the exception of age where ANOVA was used F-statistics is reported. Fisher’s Exact Test was used when frequency was lower than 5; thus no df or chi-square statistics are presented

## Discussion

Despite the road safety measures implemented in the region, there was a higher incidence of seriously injured road users in 2014 than in 2003, both in rural and urban areas, but the incidence has increased substantially more in urban areas. These findings correspond to the road safety measures implemented in the region, since fewer measures were implemented in urban areas.

### Decreased and increased incidence for car occupants on rural roads

During the study period, the incidence of injuries among car occupants on rural roads decreased on national roads by 26.5% but increased on regional roads by 55.0%. The decrease on national roads occurred despite the fact that national roads are responsible for 69% of transports on rural roads (Table 1). However, given that most road safety measures have been implemented on national roads, it is likely that these measures have prevented injuries for car occupants on these roads. In the region, 88.4% of the length of national roads was transformed by median barriers, speed cameras and speed revisions (Table 1). Previous Swedish studies also indicate a preventive effect of roads rebuilt with median barriers and speed cameras. In a study about effects of rebuilt roads, there was evidence for a 50–60% decrease in number of fatalities and seriously injured road users [28, 29]. When speed cameras were introduced in Sweden, it was estimated that they would reduce fatalities and serious injuries by 25% [30]. Several studies have been made globally about the effects of speed cameras, but they vary with location and mode of use [31]. On the other hand, fewer road safety measures were implemented on regional roads, where 16% of the length was transformed by speed reductions of 10 km/h [32]. The limited road safety measures implemented on regional roads may have contributed to the increase of seriously injured car occupants found in this study. A study about road safety effects of speed revision in Sweden reports that the number of seriously injured road users was basically unchanged on rural roads [33]. Thus, the reduced speed limits may not have been effective enough to reduce the number of seriously injured car occupants on regional roads.

Another explanation of the increased incidence on regional roads is that this increase may reflect an increase in road traffic. During the study period, vehicle kilometres on Swedish roads increased by 8.9%, but most of the increase in number of vehicle kilometres took place before the speed revision of 2009 [34]. There is also a study that reports higher fatality and serious injury rates on rural roads with low traffic density (< 2000 vehicles per day) than on roads with a higher density [35].

### Increased incidence for unprotected road users in urban areas

In urban areas, although the incidence for car occupants decreased between 2003 and 2014, the incidence for unprotected road users doubled during the study period, though more on tracks and pavements than on roads. Road safety measures in urban areas consisted of building bumps and roundabouts, and making speed limit revisions from 50 to 40 km/h or even to 30 km/h. The implementation of 30 and 40 km/h varies between municipalities in the region and is still ongoing [14]. Although these road safety measures in urban areas may have contributed to reducing serious injuries among car occupants, it seems that they have not influenced the safety of unprotected road users, given the increased incidence for this group. On the other hand, the increase was particularly strong for serious injuries on tracks and pavements where no road safety measures were conducted during the period. Vision Zero recommends separation between motor traffic and unprotected road users [11, 36], but still the incidence has increased more beside the road than on the road. One cause may be an increase in active mobility in RV. Nationally the distance walked increased during the period from 2.8 to 3.5 billion km and by bicycle from 1.8 to 2.4 billion km [37].

Of seriously injured cyclists and pedestrians in urban areas in RV 72 and 88% respectively were older than 45 years [38]. Road users injured in Non-Vision Zero Areas were on average 5.4 years older than those injured in Vision Zero areas. Of these, 60% were cyclists and pedestrians mostly in urban areas. An increased number of people are living in urban areas all over the world, and populations are aging as a result of increased life span and the baby-boom generation of 1940s. Many of them are unprotected road users prone to be injured in single crashes [5, 19, 39, 40].

### Areas transformed by Vision Zero road safety measures

Only about one-fifth of all serious injuries between 2003 and 2014 occurred in Vision Zero areas. Most of them occurred on rural roads where there is evidence of a decreased number of fatalities and serious injuries because of road safety measures such as median barriers and speed cameras.

Nearly all crashes in transformed urban areas took place on roads with a speed limit of 30 km/h. In accordance with the Vision Zero policy, unprotected road users should not be exposed to vehicles at speeds exceeding 30 km/h [11], but there are studies reporting that even this limit can be too high to prevent serious injuries to pedestrians and cyclists in crashes [41–43]. More cyclists than pedestrians are seriously injured in crashes at speeds of < 30 km/h [41, 42]. The speed limit 30 km/h was introduced in urban areas as early as 1998, but 40 km/h instead of 50 km/h has only been possible since 2010. Great variations in speed increase the probability of crashes and serious personal injuries [26].

### Public health implications

Despite efforts to reduce serious injuries by means of Vision Zero measures, a doubling of their incidence in urban areas occurred over 12 years, with pedestrians and cyclists being particularly affected. In Sweden, as in many other countries, the state promotes active mobility, both for the sake of public health and to contribute to a sustainable lifestyle for society [6]. There are conflicts, especially in urban areas, between the goals of injury prevention and promoting health through more active mobility. In a review of studies about the health impact of increased levels of active mobility, 14 studies estimated more fatalities and injuries while six studies estimated decreases of fatalities and injuries. The conclusion of the study is that active mobility provides net health benefits overall [44]. The work with implementing road safety policies hopefully aims to increase these benefits. Findings from this study may possible be extrapolated to the 17 other regions that have a larger central municipality together with a number of smaller municipalities. In the three metropolitan regions in Sweden there are more lanes for cycling on the roads.

### Strengths and limitations

The data in this study are analysed using an extended concept of mobility which includes types of injuries receiving less attention in road safety management such as pedestrians in single crashes [45]. The national indicator for seriously injured road users is furthermore a calculated value for seriously injured persons with a disability of 1% or more [46]. To calculate a corresponding value for a municipality or a region implies greater uncertainty than on national level. Instead this study uses a definition of serious injury that is linked to a scale constructed and used to determine outcome of injuries and is nearly identical to the definition recommended by EU and International Transport Forum (MAIS3+) [4, 25, 47]. Different definitions of serious injury may complicate the work with target fulfilment.

STRADA is a new registry and therefore the study uses data only from 2003, when data from health care and police were collected in the same system [48]. Trend analysis handles the variation in values during the period, but some values are extreme cases as the value for car occupants on national roads in 2006 linked to a bus crash. Values for unprotected road users on tracks and pavements 2010 and 2011 are related to unusually long periods of winter weather (Figs. 1, 2 and 3) [49]. Data from more regions or a longer period would probably have resulted in more statistical power.

Possible confounders such as changes in the number of vehicles or unprotected road users in the traffic that could have contributed to the decrease of seriously injured car users or the increase of seriously injured unprotected road users are not included in the analysis since such information is not available. The bivariate nature of the comparisons between rural and urban areas and between vision zero areas and non-vision zero areas also limit interpretations since no confounders are controlled for. Further, non-stationarity and autocorrelations were not considered when showing the linear regressions for time series due to a small number of time points (12 time points). This is also a limitation when interpreting the results of this study.

## Conclusion

Despite implementation of road safety measures in the region, the incidence of seriously injured road users doubled in urban areas during 2003–2014, while the incidence decreased for car occupants on transformed national roads in rural areas. In urban areas, more people were seriously injured on pavements and tracks for cycling and walking than on the roads where Vision Zero had been implemented.

The reduction of the incidence for car occupants in the region may not be adequate to fulfil the national target. More must be done, especially in the urban areas where more active mobility is desired.

## Data Availability

Data from the registry STRADA cannot not be publicly available due to Swedish legislation. The responsible authority is the Swedish Transport Agency. This authority is responsible for obtaining consent from research subjects before data is entered into the database. The information system STRADA is covered by the law with supplementary provisions to the EU Data Protection Regulation (SFS 2018: 218) and the Public and Privacy Act (SFS 2009: 400). Data is only available for researchers, who meet the criteria for access to the STRADA registry.

## Abbreviations

AIS: Abbreviated injury scale
ISS: Injury Severity Score
NVDB: National Road Database
RV: Region Västmanland
STRADA: Swedish Traffic Accident Data Acquisition
